# Diagnosis and Treatment of NMO Spectrum Disorder and MOG-Encephalomyelitis

**DOI:** 10.3389/fneur.2018.00888

**Published:** 2018-10-23

**Authors:** Nadja Borisow, Masahiro Mori, Satoshi Kuwabara, Michael Scheel, Friedemann Paul

**Affiliations:** ^1^NeuroCure Clinical Research Center, Charité – Universitätsmedizin Berlin, Corporate Member of Freie Universität Berlin, Humboldt-Universität zu Berlin, and Berlin Institute of Health, Berlin, Germany; ^2^Department of Neurology, Graduate School of Medicine, Chiba University, Chiba, Japan; ^3^Department of Neuroradiology, Charité – Universitätsmedizin Berlin, Corporate Member of Freie Universität Berlin, Humboldt-Universität zu Berlin, and Berlin Institute of Health, Berlin, Germany; ^4^Experimental and Clinical Research Center, Max Delbrueck Center for Molecular Medicine and Charité – Universitätsmedizin Berlin, Berlin, Germany

**Keywords:** neuromyelitis optica, aquaporin-4 antibodies, MOG-encephalomyelitis, diagnostic criteria, immunosuppressive treatment

## Abstract

Neuromyelitis optica spectrum disorders (NMOSD) are autoantibody mediated chronic inflammatory diseases. Serum antibodies (Abs) against the aquaporin-4 water channel lead to recurrent attacks of optic neuritis, myelitis and/or brainstem syndromes. In some patients with symptoms of NMOSD, no AQP4-Abs but Abs against myelin-oligodendrocyte-glycoprotein (MOG) are detectable. These clinical syndromes are now frequently referred to as “MOG-encephalomyelitis” (MOG-EM). Here we give an overview on current recommendations concerning diagnosis of NMOSD and MOG-EM. These include antibody and further laboratory testing, MR imaging and optical coherence tomography. We discuss therapeutic options of acute attacks as well as longterm immunosuppressive treatment, including azathioprine, rituximab, and immunoglobulins.

## Introduction

Neuromyelitis optica spectrum disorders (NMOSD) are rare chronic inflammatory central nervous system diseases distinct from multiple sclerosis (MS). The French term “neuro-myélite optique aiguë,” which may be translated as “neuromyelitis optica acuta” was first used by Devic in 1894 (1, 2). In the majority of patients with NMOSD, autoantibodies (Abs) against the astrocyte aquaporin-4 (AQP4) water channel are detectable and patients typically suffer from recurrent attacks of severe optic neuritis or/and myelitis (3–7). In rarer cases, brainstem and brain involvement e.g., area postrema syndrome or diencephalic syndrome can occur (8, 9). Patients also frequently suffer from burdensome symptoms like pain, headache, depression, fatigue, and sleep disorders (10–14). Despite treatment, recovery from attacks is often incomplete and disease remission rarely occurs (15, 16). Thus, in relapsing NMOSD, which account for approximately 80–85% of cases, neurologic deficits frequently accumulate during the disease course. Patients without long-term immunosuppressive therapy have a worse prognosis with a higher mortality rate (17). Disease onset ranges between 4 and 88 years with a mean age at onset of 39 years (18–21). Women are disproportionately more often affected and, particularly in AQP4-seropositive patients, female to male-ratio can reach up to 10:1 (19, 22, 23). In 20–30% of patients, depending on the assay used, AQP4-Abs are not detectable (24, 25). Whether AQP4-Ab positive and AQP4-Ab negative diseases are varieties of the same disorder or rather reflect different disease entities is a topic of ongoing research (26–28).

Recently, various publications described the detection of serum-Abs against myelin-oligodendrocyte-glycoprotein (MOG) in AQP4-Ab negative NMOSD patients including pediatric cohorts and few patients with MS (29–41). In the past, MOG-Abs were particularly described in acute disseminated encephalomyelitis (ADEM), an inflammatory CNS disorder that, if it has an pediatric onset, is mostly monophasic and has a favorable outcome in the majority of cases (42, 43). MOG is a glycoprotein localized on the surface of the myelin sheath as well as of the cell body and processes of oligodendrocytes (44, 45). According to the revised 2015 NMOSD diagnostic criteria (46), diseases with or without evidence of AQP4-Abs as well as disorders with MOG-Abs can be assigned to the NMO spectrum. Although there are numerous overlaps in clinical presentation and imaging findings with NMOSD with and without AQP4-Ab, MOG-Ab-associated disease is more and more considered a disease entity in its own (47). Previous studies on NMOSD might have included patients with MOG-Abs and therefore overlapping features could have been reported in these studies. Various terms are used to describe the disease such as “MOG-antibody related disorder,” “MOG-associated disease,” “MOG antibody disease,” “MONEM” or “MOG-encephalomyelitis (MOG-EM)”(40, 47–50). Hereafter, we use the term “MOG-EM,” as it reflects the relevant symptoms of the disease and is used in several recent publications, e.g., (49). Although ADEM can also be accompanied by MOG-Abs (51), in this manuscript we do not regard MOG-Ab positive patients with ADEM-phenotype as part of the “MOG-EM” due to their distinct clinical characteristics. To date, the relevance of MOG-Abs and their nosologic categorization is a topic of current discussion and under further investigation (47, 52, 53).

To give an overview on diagnosis and treatment recommendations in NMOSD and MOG-EM, we here describe our own clinical experiences and give a review on the current literature using the Pubmed online database. We used the search terms “neuromyelitis optica,” “neuromyelitis optica spectrum disorder,” “MOG,” aquaporin-4 antibodies,” “MRI,” “diagnostic criteria,” “therapy,” and combinations of these. To find all relevant publications, we did not restrict the year of publication; however, most reports originate from the last 5 years.

## Diagnosis

In NMOSD and MOG-EM, most common symptoms are optic neuritis and longitudinally extensive transverse myelitis (LETM). Signs of brainstem affection like persistent hiccup, nausea or vomiting should explicitly be asked for as they are often attributed to other reasons and are therefore not reported spontaneously by the patient. Rarer clinical manifestations of NMOSD comprise narcolepsy, acute diencephalic syndrome or muscle affection (54, 55), while in MOG-EM extraneural involvement such as reversible paraspinal muscle hyperintensity have been described, as well as MOG-Abs in combined central and peripheral demyelination syndromes (56, 57).

Like NMOSD, MOG-EM can affect optic nerve, spinal cord, and brainstem. However, some studies showed histopathological differences between NMOSD and MOG-EM (58, 59). AQP4-Abs bind to water channels located on astrocytes, whereas MOG-Abs target myelin-forming oligodendrocytes (53). Both types of antibodies may lead to disturbances of the integrity of blood brain barrier and to CNS inflammation (53, 60). However, while inflammation in MOG-EM primarily results in demyelination, demyelination in NMOSD seems to be a secondary phenomenon following astrocytic damage (61, 62).

In patients with AQP4-Abs, the most frequent symptoms at onset are optic neuritis in 37-54% of the patients, and LETM in 30–47% of the patients (26, 63, 64). In patients with MOG-Abs, optic neuritis was the first clinical manifestation in 33–64% whereas myelitis occurred in 18–33% of the patients as initial symptom (33, 48, 65). Also during the further course of the disease, optic neuritis seems to be more frequent in MOG-EM than in NMOSD with myelitis being less common (29, 66). However, in population-based ON studies and unselected cohorts of patients with ON, both the prevalence of AQP4-Abs and MOG-Abs is low (67–69). In MOG-EM, cases of encephalitis and seizures were described whereas these symptoms are rare in NMOSD (70–72). MOG-EM differs from NMOSD in further clinical characteristics e.g., in gender ratio and age at onset. In (relapsing) NMOSD, up to 90% of the patients are female, whereas the proportion of male patients in MOG-EM ranges from 43 to 63% (22, 26, 29–31, 73). The published mean age at onset ranges from 27 and 37 years in patients with MOG-EM (29–31, 73) and between 30 and 46 years for patients with NMOSD (19, 26, 29–31, 73). At onset, patients with MOG-Abs are more likely to suffer from simultaneous or rapidly sequential optic neuritis and LETM compared to patients with AQP4-Abs (31). In AQP4-Ab positive NMOSD, most patients (80–90%) have a relapsing disease course (26, 73, 74). In MOG-EM, monophasic disease course is considered to be more frequent, however, the duration of follow-up and a referral bias might have influenced these results (33, 73–76). Some studies showed lower disability outcomes, measured by the Expanded Disability Status Scale (EDSS), in MOG-EM than in NMOSD, suggesting a presumably more favorable prognosis (29–31, 73). However, long-term data from MOG-EM are scant. Whereas spinal cord lesions frequently affect cervicothoracic segments in NMOSD, they tend to be localized in thoracolumbar parts of the spinal cord including the conus in MOG-EM (29, 31). Table 1 summarizes the epidemiological and clinical features in NMOSD and MOG-EM.

**Table 1:** Epidemiological and clinical features in NMOSD and MOG-EM.

	**AQP4-Ab positive NMOSD**	**MOG-EM**
Mean age at onset [range]	40–46 years (26, 31, 73)	27–37 years (30, 73)
Female to male ratio [range]	7.2:1–10:1 (26, 29, 31, 73)	1:1.6–1.3:1 (29–31)
Median EDSS at last follow-up [range]	4.0–5.8 (29, 31, 73)	0–1.5 (29, 31, 73)
Frequency of coexisting autoimmune diseases	16–45% (31, 73)	6–11% (31, 73)
Localization of optic nerve lesions	orbital, chiasm (77)	orbital, canalicular, intracranial (77)
Features of optic neuritis	OCT: prominent RNFL thinning (77)	Severe optic nerve swelling at onset (77); frequently simultaneous or rapidly sequential optic neuritis and LETM (31)
Localization of spinal cord lesions	Cervical, thoracic (29)	Thoracic, lumbar (29), involving conus (31)
MRI brain lesions	More frequently lesions in medulla oblongata and area postrema (65)	More frequently ADEM-like brain lesions, deep gray matter lesions (31), lesions in pons, thalamus (65)

### Antibody diagnosis

A central component of diagnostics in NMOSD and MOG-EM is the detection of Abs in serum. AQP4-Abs were firstly described in 2004 and made it possible to differentiate NMOSD from MS (78). The best detection rates are provided by cell-based assays (CBA) (24, 32, 79, 80). In NMOSD, the sensitivity of these assays ranges between 80 and 100%, whereas specificity varies between 86 and 100% (24). Contrarily, enzyme-linked immunosorbent assays (ELISA) may lead to false-positive results and should not be used as sole method (81–83).

Specific antibodies against MOG are detectable in pediatric patients with acute disseminated encephalomyelitis (ADEM) (84–86). In MS patients, MOG-Abs were described for the first time at the beginning of the 1990s (87). Further studies confirmed these findings (88–90). Later, MOG-Abs were found in AQP4-Ab negative patients with clinical symptoms of NMOSD (91, 92). Like in NMOSD, CBA are the current gold standard to detect MOG-Abs (39, 49). Formerly used assays had a low MOG specificity, which led to high rates of false positive results (39, 49). Therefore to date, cell-based assays targeting at full-length human MOG and the use of IgG1-specific secondary antibodies is highly recommended to avoid cross-reactivity with IgM and IgA antibodies (39, 49).

AQP4- and MOG-serostatus and Ab-level may change during the disease course. In patients with suspected NMOSD or MOG-EM without initial evidence for seropositivity, further Ab-testing may be required during the course of the disease, especially during acute attacks and intervals without treatment. AQP4-Abs usually stay detectable during remission, although the titer may be lower with immunosuppression (some patients even seroconvert to negative over time) and during acute attacks (93). In MOG-EM, approximately 80% of patients with evidence of MOG-Abs during acute attack remained seropositive during remission (33). However, the rate was lower with only 50% of patients remaining seropositive in a study from Korea (76). As in AQP4-Ab positive NMOSD, MOG-Ab serum titers are significantly higher during acute attack than during remission (33). In some studies, Ab-titers were associated with relapses and treatment status (32, 93, 94). However, the level of AQP4-titer does not seem to be predictive for long term disease course (95), and AQP4-Ab serostatus is not predictive of response to immunotherapy (96). Testing of patients with progressive MS for MOG antibodies is not warranted under most circumstances (97).

Testing CSF for AQP4- or MOG Abs is not routinely recommended as it does not seem to provide an additional benefit for diagnosing NMOSD or MOG-EM (98, 99). AQP4-Abs in CSF can be detected in only 70% of Aqp4-Ab seropositive patients and in none of the AQP4 seronegative patients (100). Like AQP4-Abs, MOG-Abs are produced mainly extrathecally and are therefore less frequent in CSF than in serum (32).

Comorbidity with other autoimmune disorders is frequent in NMOSD patients (101–105). Therefore, further tests for autoantibodies should comprise Abs associated with rheumatologic diseases e.g., ANA, ANCA, Anti-ds-DNA-Abs, and lupus anticoagulant. If there are clinical signs in anamnesis or examination, Ab testing for myasthenia gravis, coeliac disease, or paraneoplastic disorders should be performed (101, 106–110). In MOG-EM, the frequency of coexistent autoimmune diseases seems to be lower than reported for AQP4-Ab positive patients (33, 66).

### Further laboratory diagnosis

Other laboratory tests are recommended to diagnose coexisting autoimmune disorders and to exclude other differential diagnoses. Next to routine laboratory tests, this includes differential blood count, blood sedimentation, folic acid, and vitamin B12 (111). To exclude sarcoidosis which is a relevant differential diagnosis as it can also manifest with optic neuropathy or myelopathy (112, 113), tests on hypercalcemia and hypercalciuria, interleukin-2-rezeptor (sIL-2 R), and angiotensin-converting enzyme (ACE) should be performed (112, 114).

Analysis of cerebrospinal fluid (CSF) might be helpful to exclude other diagnoses, especially to differentiate between NMOSD/MOG-EM and MS. In NMOSD, white cell counts were elevated in up to 50% of the patients, especially during acute attack, and in approximately 10% of the patients CSF-restricted oligoclonal IgG bands (OCB) can be detected (73, 100). Increased CSF/serum albumin ratio as a marker of dysfunction of blood brain barrier was found in 51% of NMOSD patients (100).

In MOG-EM, elevated white cell counts were found in 25–70% of the patients, whereas there was no differentiation between tests during acute attack and remission. (33, 66, 73). Like in NMOSD, OCB were detected in 10% of the MOG-EM patients and CSF/serum albumin ratio was elevated in 32% (33, 66, 73).

### Magnetic resonance imaging

Next to the AQP4-Abs, MRI is an essential element to diagnose NMOSD. It helps to differentiate NMOSD from MS and other CNS disorders (115).

Spinal cord imaging was already included in the 2006 NMO diagnostic criteria (116). These criteria require MRI spinal cord lesion extending over ≥3 vertebral segments (116). However, in 15 percent of myelitis attacks, spinal cord lesions do not extend over ≥3 vertebral segments which may lead to misdiagnosis or delayed diagnosis of NMOSD (117). Typical NMOSD lesions are located centrally in the spinal cord and involve more than the half of spinal cord cross-section area (118). It was suggested by Yonezu et al.(119) that “bright spotty lesions” are characteristic for NMOSD and might reflect microcystic defects of the spinal cord (113). The specificity of this sign however still needs to be confirmed in further studies. The interval between clinical symptoms and the MRI is influencing the MRI presentation of LETM lesions. They may not be present from relapse onset and may change into multiple short lesions or into spinal cord atrophy during the disease course (120, 121). Hence, there is the risk miss a typical MRI presentation of the LETM when the MRI is performed too early or too late (72). Other causes for longitudinally extensive spinal cord LETM lesions include sarcoidosis or spondylotic myelopathy or rarely MS and need to be considered (112, 122, 123). In addition longitudinally extensive myelitis lesions were recently described in patients with symptoms of meningitis, encephalitis and/or myelitis that were tested positive for glial fibrillary acidic protein (GFAP)-IgG (124–126).

Brain MRI at first presentation often shows no lesions which has been the reason to define normal brain MRI as one NMO diagnostic criterion in 2006 (116). However, more recent studies showed that the presence of cerebral lesions is not uncommon in the clinical course of NMOSD (127–129). Hence, the NMOSD 2015 diagnostic criteria have incorporated findings of cerebral MRI and define NMOSD-typical brain lesions (46). These lesions can be located at the periependymal surfaces of the third and fourth ventricle, in the area postrema, corpus callosum, hypothalamus or thalamus (130–132). In addition, subcortical or deep white matter lesions are possible. Meningeal enhancement has been reported in some cases, although this does not appear to be a very frequent imaging finding in NMOSD (133). Orbital MRI may show increased T2 signal and gadolinium enhancement of the optic nerve as signs of an optic neuritis. This can be helpful to diagnose MOG-EM or NMOSD in patients without AQP4-Abs (46, 77, 131, 134, 135). Chiasmal involvement is more common in AQP4-NMOSD than in MOG-EM (134).

A study by Ramanathan et al. showed no MRI brain lesions in a large proportion of MOG-EM patients (66). Conversely, other authors found supra- and infratentorial MRI abnormalities in 40–50% of the patients (33, 65). Brain imaging allows to distinguish MOG-EM from MS, but shows many overlaps with AQP4-Ab NMOSD (136–138). Moreover, a relevant number of patients show pathologic findings in MRI of optic nerve and spinal cord, comparable to NMOSD patients (33, 74). However, one study revealed a more frequent occurrence of optic nerve head swelling and retrobulbar affection of the optic nerve in MOG-EM compared to NMOSD (134).

**Figure 1:**
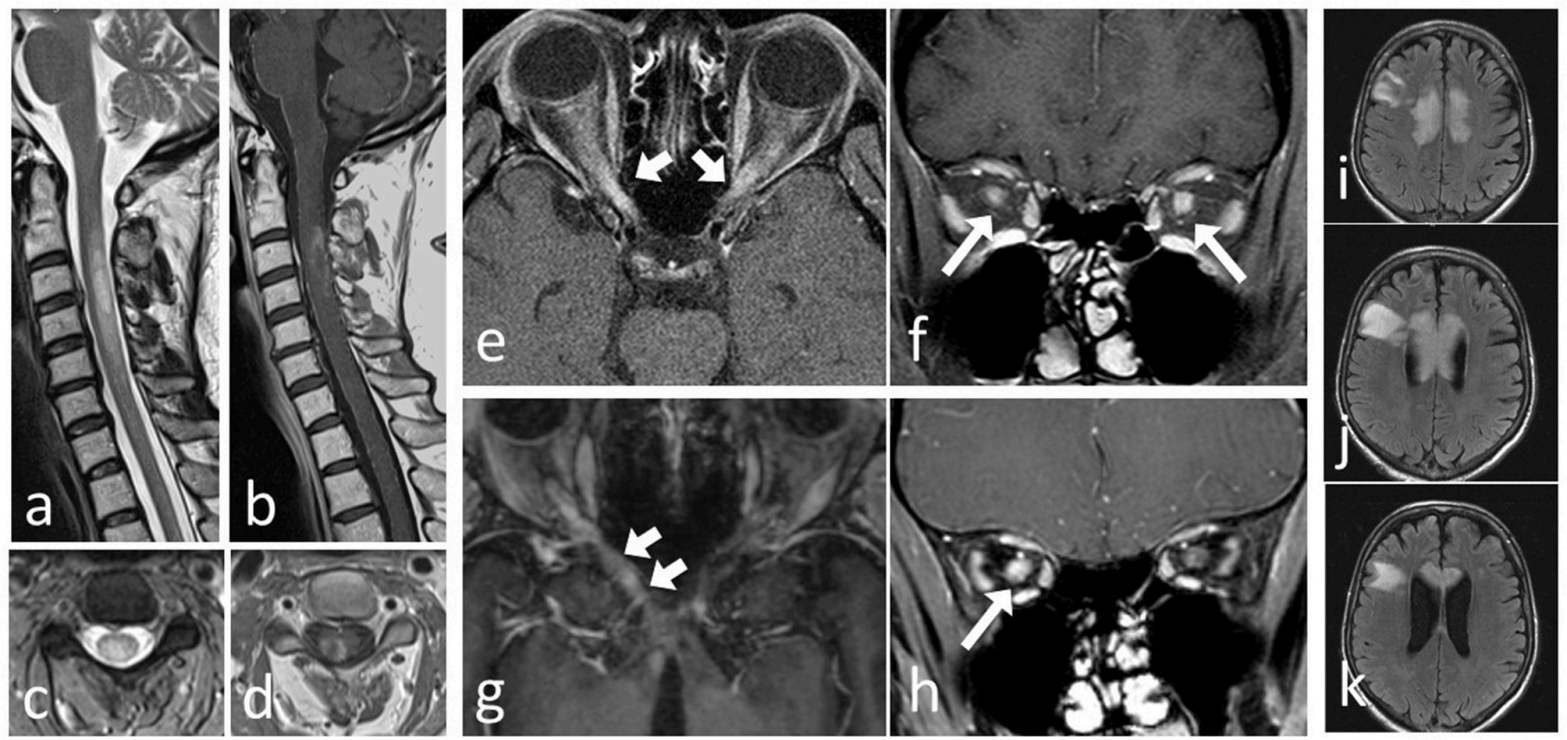
MRI of patients with AQP4-Ab positive NMOSD and patients with MOG-EM. Severe cervical LETM in a NMOSD AQP+ patient: **(a)** T2 sagittal and **(b)** T2 axial of a cervical myelon lesion with ring Gd-Enhancement and T1 hypointense center in **(c)** T1+Gd sagittal and **(d)** T1+Gd axial. Bilateral opticusneuritis in a MOG-EM patient **(e)** T1+Gd axial and **(f)** T1+Gd coronar. Unilateral optic neuritis with chiasmal involvement in a NMOSD AQP4+ patient: **(g)** T1+Gd axial and **(h)** T1+Gd coronar. Tumefactive lesion involving the corpus callosum in a NMOSD AQP4+ patient **(i–k)** T2 axial.

Figure 1 shows MRI features of NMOSD and MOG-EM. Studies investigating non-conventional MR imaging in NMOSD will not be reviewed further as they currently lack implications for clinical management (139–141).

### Optical coherence tomography

Optical coherence tomography is an interferometric technique using near infra-red backscattered light to generate high resolution images of the retina and its various layers, that is increasingly applied in various neuroimmunological disorders (142–148). OCT displays severe damage to the retinal nerve fiber layer and the ganglion cell layer following attacks of optic neuritis in both AQP4 NMOSD and MOG-EM that correlates with visual function and quality of life (149–158). It is currently a matter of debate if retinal damage following optic neuritis is equally severe in AQP4-NMOSD and MOG-EM (75, 77, 152, 159–162) and to which extent structural retinal alterations occur in NMOSD independently of optic neuritis attacks (143, 157, 163–167). Although the utility of OCT in patient management requires further investigation, it may help quantify the extent of structural retinal damage following optic neuritis attacks and thus hopefully inform treatment decisions (168–170), and support differential diagnosis in the near future.

### Diagnostic criteria

Current NMOSD diagnostic criteria were published by the International Panel for NMO Diagnosis in 2015 (46) and were aimed at taking recent advances in the field following the 2006 Wingerchuk criteria into consideration (116). They differentiate between NMOSD with AQP4-Abs and NMOSD without AQP4-Abs or unknown AQP4-Ab status.

In the case of positive AQP4-Ab status, one of the following clinical core symptoms is required:

1. Optic neuritis
2. Acute myelitis
3. Area postrema syndrome: episode of otherwise unexplained hiccups or nausea and vomiting
4. Acute brainstem syndrome
5. Symptomatic narcolepsy or acute diencephalic clinical syndrome with NMOSD-typical diencephalic MRI lesions
6. Symptomatic cerebral syndrome with NMOSD-typical brain lesions

NMOSD-typical brain lesions may involve the dorsal medulla, especially the area postrema, the periependymal surfaces of the third or fourth ventricle, the hypothalamus, thalamus, the corpus callosum, cerebral peduncles, and the internal capsule. Moreover, subcortical or deep white matter lesions and corticospinal tract lesions are possible. Alternative diagnoses e.g., MS, sarcoidosis, infectious or neoplastic diseases have to be excluded.

In patients without evidence of AQP4-Ab two of the above mentioned core clinical characteristics are necessary for NMOSD diagnosis. At least one of these core clinical characteristics has to be ON, LETM or area postrema syndrome. Moreover, supportive characteristics in cerebral, spinal cord or optic nerve MRI are required. These are

- normal brain MRI or long optic nerve lesions with increased T2 signal or gadolinium enhancement of the optic nerve or the chiasm in patients with ON,
- spinal cord MRI lesion or focal spinal cord atrophy extending over ≥3 segments in patients with myelitis and
- lesions involving dorsal medulla oblongata/area postrema in patients with area postrema syndrome
- periependymal brainstem lesions in patients with acute brainstem syndrome.

Using the 2015 instead of the 2006 criteria led to a significant increase in the number of patients diagnosed with NMOSD (138, 171, 172)

For MOG-EM, to date, no evidence based diagnostic criteria exist. However, NMOSD 2015 diagnostic criteria allow to include cases of NMOSD associated with other specific autoantibodies (46).

## Treatment of acute attacks

In NMOSD as well as in MOG-EM, acute attacks are usually treated with 1,000 mg intravenous methylprednisolone (IVMP) for 3–5 days. Jarius et al. showed complete or almost complete recovery in 50% of IVMP treated MOG-EM attacks (33). In NMOSD, IVMP led to complete recovery in 17–35% of the attacks (15, 173). In case of poor response, treatment escalation with 2,000 mg IVMP may improve outcome, for further therapy escalation plasma exchange (PLEX) or immunoadsorption are possible (15, 173–175). PLEX and immunoadsorption did not show a difference in their efficacy in the therapy of NMOSD attacks (176). They can also be used as first-line therapy (in particular in myelitis attacks) if response to methylprednisolone during previous attacks was poor. An early initiation of PLEX seems to improve the clinical outcome (176, 177).

## Preventative immunosuppressive therapy

Attacks in NMOSD as well as in MOG-EM are often characterized by severe neurologic deficits with poor recovery. Frequently, a relevant disability persists after an attack. However, there are indications that MOG-EM has a less severe course than NMOSD and relapse risk depends on Ab status (30, 65, 66). In some patients with evidence of MOG-Abs, seroconversion to an Ab- negative status may occur during the disease course (30, 32, 76).

There is increasing evidence that immunosuppressive therapy is essential to reduce disease activity and to avoid further attacks. However, to date no placebo controlled trial has been published and only one open randomized clinical trial has been performed (178). Thus, the current treatment paradigm is based on case series, (retrospective) observational studies as well as expert opinion. Hereafter, we describe the to-date used treatments in NMOSD and MOG-EM (179).

### Low dose prednisone/prednisolone

Low dose oral corticosteroids are used in many neurologic diseases. Oral prednisone/prednisolone can be given subsequent to attack therapy with IVMP in decreasing dose levels and as comedication during the first months of azathioprine (AZA) or mycophenolate mofetil (MMF) treatment until these drugs exert their full efficacy. Possible side effects are weight gain, hypertension, thrombosis, osteoporosis, fungal and viral infections, hyperglycemia, gastritis and peptic ulcer, psychiatric disturbances and a Cushing syndrome (180).

Data on long-term treatment with oral prednisone/prednisolone in NMOSD are limited. A few studies could show a decrease in ARR by low dose steroid therapy (181, 182). Moreover, it is known from treatment experiences with AZA that additional oral prednisone is effective to reduce disease activity during the first 3–6 months until AZA reaches its full efficacy.

In MOG-EM, low treatment failure rates were achieved with oral prednisone (66). The occurrence of relapses during tapering or after cessation of subsequent oral prednisone after IVMP attack treatment supports the beneficial effects of corticosteroid therapy in MOG-EM (33), at least in patients with persistence of MOG Abs (66). However, due to the known side effects and the existence of other treatment alternatives, a long-term therapy with low dose prednisone should be critically weighed.

### Azathioprine

AZA is a purine analog, acts as antimetabolite and inhibits the differentiation of lymphocytes. Thereby it has antiproliferative and immunosuppressive effects. It is administered in a dose of 2–3 mg/kg body weight per day and reaches its full effectiveness after 3–6 months. During the initial period, additional oral prednisone [1 mg/kg/day] is necessary and can be slowly tapered when AZA becomes fully effective.

The most important side effect is a bone marrow depression with anemia, leuko- and/or thrombopenia. The risk of bacterial, viral or fungal infection is increased. Moreover, elevation of liver enzymes, nausea or emesis can appear. Rare side effects especially after long treatment duration include malignomas e.g., of the skin, and a progressive multifocal leukoencephalopathy (PML). Furthermore, add-on therapy with prednisone enhances the risk of side effects, like a diabetogenic metabolic state, thrombosis or psychiatric symptoms.

Patients with a congenital deficiency of thiopurinmethyltransferase (TPMT), an enzyme responsible for metabolisation of AZA, have a high risk of bone marrow depression. Therefore, it is recommended to test for TPMT-deficiency in patients with pronounced deterioration of blood count after initiation of AZA-therapy.

A recently published prospective randomized controlled trial compared the efficacy of AZA and rituximab (RTX) in NMOSD. It showed a significant decrease in mean ARR from 1 to 0.51 and a decrease in mean EDSS from 2.40 to 1.95 by AZA (178). 54% of the patient treated with AZA became relapse free after 1 year (178). A prospective study including 77 NMOSD patients (183) and other retrospective studies (181, 184–186) showed comparable results.

In a study by Jarius et al. 14 out of 17 MOG-patients (82%) suffered from at least one attack while treated with AZA (33). Attacks occurred mainly in patients that were not co-treated with oral prednisone and during the first 6 months. This highlights the need for co-treatment with oral prednisone until AZA reaches its full efficacy.

### Rituximab

RTX is a monoclonal Ab directed against the surface molecule CD20 on B-lymphocytes. RTX leads to a depletion of CD20+B-lymphocytes, which act as precursor cells of antibody producing plasma cells (187). A thereby triggered reduction of antibody formation is presumably the RTX mechanism of action.

The most frequently used dose regimen is the intravenous administration of each 1,000mg with an interval of 2 weeks followed by 6-monthly dosages of 1,000mg (179, 188). Alternatively, initially 375 mg/m^2^ body surface every week over a period of 4 weeks can be administered. As an alternative to a fixed dosage regimen every 6 months, monitoring of CD19+/CD20+ B-lymphocytes and administration of RTX in the case of reconstitution of these cells is possible (189, 190). Another option is the administration of RTX depending on monitoring of CD27+ memory B-cells which might in some cases allow to lower the cumulative RTX dose (191). An evidence that one of these regimens has therapeutic superiority over the other does not exist to date.

Before first administration, active infections like tuberculosis or hepatitis B have to be excluded (192). An update of vaccination status and anti-pneumococcal vaccination is recommended (192).

Side effects include infusion-related symptoms like pruritus, headache, rash or fever. To reduce the risk of these symptoms, a premedication with an analgesic/antipyretic and an antihistamine is recommended. The risk of infections and severe skin reactions like the Lyell-syndrome or the Stevens-Johnson-Syndrome is elevated. Cardiac symptoms e.g., arrhythmia or cardiac insufficiency were reported (193). Moreover, neurologists must be aware of hypogammaglobulinemia that may occur with long-term RTX treatment (194).

In 2005, an open label study described for the first time a significant reduction in disease activity in eight NMO patients treated with RTX (195). Since then, an increasing number of patients was treated with RTX. However, to date only a few prospective studies investigating the effect of RTX on NMOSD exist. The above mentioned study by Nikoo et al. showed a reduction of the ARR by 83 percent as mean ARR decreased from 1.30 to 0.21 (178). Mean EDSS decreased from 3.55 to 2.56. Other prospective and retrospective trials found significant reductions of ARR to values between 0.1 and 0.46 in adult and pediatric patients treated with RTX (181, 196–202). A further overview on efficacy and safety profile of RTX in NMOSD is given in the topical literature (203–205).

AZA and RTX are the most frequently used immunosuppressants in NMOSD. With regards to ARR and EDSS, comparison studies between both drugs seem to suggest a superiority of RTX compared to AZA (178, 184). Thus, currently RTX seems to be the most effective treatment in NMOSD, although some studies describe a rebound in disease activity shortly after RTX induction (199, 206). Treatment effect does not seem to depend on AQP4-serostatus (96).

In MOG-EM, treatment with RTX led to a decline in relapse rate in only 3 out of 9 patients (33). Most of the attacks occurred shortly after RTX infusion. Some authors recommend RTX as second-line-therapy if preventative treatment with low-dose prednisone or monthly intravenous immunoglobulins (IVIG) is not effective (66). In patients with myelitis, RTX is recommended from an earlier stage as a myelitis often leads to severe residual deficits (66). Whether RTX is indeed less effective in MOG-EM than in NMOSD has to be analyzed in further studies (207).

### Mycophenolate mofetil

MMF is an immunosuppressant that inhibits the inosine monophosphate dehydrogenase. Thereby, the synthesis of guanosin nucleotide and subsequently, the proliferation of B- and T-lymphocytes is inhibited. The administered daily dose ranges between 750 and 3,000 mg/d (179, 208, 209).

The most common side effects are leucopenia, diarrhea, vomiting and sepsis. The risk of malignomas can be increased especially if MMF is combined with other immunosuppressants.

A retrospective observational study investigated the effect of MMF in NMOSD and MOG-EM. 33/67 (49%) of the patients were relapse-free, in 44/53 (83%) the EDSS improved or stabilized (208). Other observational studies showed similar results with proportions of relapse-free patients between 56 and 60% in NMOSD (210, 211) In comparison to AZA, MMF showed fewer side effects with equal efficacy (211, 212). As in treatment with RTX, response to MMF does not differ in dependence on AQP4-serostatus (96).

In MOG-EM patients, a combined therapy with MMF and steroids appeared to have a positive effect; however, this effect diminished after steroid tapering (66). As MMF may take several months to reach its full efficacy, add-on prednisone should be tapered only very slowly.

### Intravenous immunoglobulins

Even less data is available for treatment with IVIG in NMOSD. A small retrospective study including six patients with NMO/NMOSD treated with IVIG 2–3- monthly showed a decrease in ARR from 0.75 to 0.15 (213). One study investigated IVIG treatment of acute NMOSD relapses (214), however, further data on preventive IVIG therapy is lacking.

In a study by Ramanathan et al, 4 out of 7 MOG-EM patients treated with IVIG were relapse-free (66). The authors recommend prophylaxis with low-dose prednisone or monthly IVIG with MMF or RTX as a next step for treating MOG-EM. Jarius et al. reported data of one MOG-EM patient who was relapse-free during 11 months of IVIG treatment and 12 months after IVIG discontinuation (33).

### Methotrexate

Methotrexate (MTX) is an analog of folate, acts as folate antagonist and inhibits the dihydrofolate reductase. Hereby it inhibits DNA and RNA synthesis and has an immunosuppressive and anti-inflammatory effect. Side effects include gastrointestinal symptoms like nausea or diarrhea, bone marrow depression and an increase of liver enzymes.

Retrospective studies in NMOSD showed a decrease of ARR between 64 and 87% (215–217). In MOG-EM, MTX led to disease stabilization in 5/6 patients (33). Therefore, MTX seems to be a treatment option in patients that do not respond to first-line-therapy or suffer from side effects of other treatments (216).

### MS immunomodulatory medication and rarer treatment options

Treatment with MS medications like interferon-beta, glatiramer acetate, fingolimod, alemtuzumab, natalizumab, and presumably also dimethyl fumarate is known to have no or even harmful effects in NMOSD (181, 218–230). Similar results were found in patients with MOG-EM which were treated with one of these drugs for suspected MS (33); however, studies on treatment effects of these drugs are even rarer than in NMOSD.

Mitoxantrone is able to significantly reduce ARR in NMOSD patients (231, 232), nevertheless, due to its cardio- and myelotoxic side effects and the availability of alternatives with fewer adverse events its use should be considered very critically (233–235). Cyclophosphamide does not seem to be effective in NMOSD (236). Data about the effects of mitoxantrone or cyclophosphamide in MOG-EM are missing.

### Ongoing studies

To date, various clinical trials are ongoing to investigate the effect of new drugs in NMOSD. A placebo-controlled clinical trial is testing the effect of inebelizumab (MEDI-551), a humanized monoclonal antibody against CD19+ B-cells on NMOSD relapse rate (237–239). The efficacy of B-cell-depleting therapy in NMOSD is well known from treatment with RTX. AQP4-Ab positive as well as AQP4-Ab negative patients with at least one relapse during the last year or with at least two relapses during the last 2 years before screening can be included in this study (237).

Another agent under investigation is eculizumab, a monoclonal antibody inhibiting the complement protein C5. There were encouraging findings from an open label study where 12 out of 14 highly active patients became relapse-free by eculizumab treatment (240, 241). A subsequent double-blind placebo-controlled phase 3 trial aiming to enroll approximately 130 patients is now in the open-label extension stage (242).

Tocilizumab, an inhibitor of the IL-6 signaling pathway, showed significant reduction of disease activity in two pilot studies including in total 15 patients with high-active NMOSD (243, 244). Moreover, it might be an option in NMOSD patients with concomitant cancer or paraneoplastic syndrome (245). To date, an open label randomized controlled trial comparing tocilizumab and AZA is recruiting patients (246). Satralizumab (SA237), a follow-on monoclonal antibody of tocilizumab, is under investigation in a placebo-controlled double-blind phase 3 study (247). Efforts to restore immune tolerance as novel therapeutic endeavor are in preparation, however, various technical and conceptual issues hamper prompt implementation in clinical trials and practice (248, 249).

Further information on ongoing or completed (pilot) studies as well as non-conventional treatment approaches, e.g., with cetirizine, regulatory dentritic cells or autologous bone marrow derived stem cells in NMOSD may be found at the website https://clinicaltrials.gov and in current literature (188, 250, 251).

## Summary

The diagnosis and treatment in NMOSD and MOG-EM require special clinical expertise. The 2015 NMOSD diagnostic criteria and the availability of antibody testing and MRI are the basis to diagnose and differentiate NMOSD or MOG-EM. Early diagnosis and initiation of adequate therapy are essential—at least in seropositive patients–to avoid disease attacks and persistent deficits. Long term immunosuppressive treatment, e.g., with RTX or AZA, has emerged to be the most effective therapies to reduce disease activity. Further therapeutic options, in particular various monoclonal antibodies are currently under clinical investigation in NMOSD.

## Author contributions

NB performed literature research and drafted the manuscript. FP, MM, MS, and SK critically reviewed the manuscript.

### Conflict of interest statement

FP serves on the scientific advisory board for Novartis; received speaker honoraria and travel funding from Bayer, Novartis, Biogen Idec, Teva, Sanofi-Aventis/Genzyme, Merck Serono, Alexion, Chugai, MedImmune, and Shire; is an academic editor for *PLoS One*, is an associate editor for *Neurology*® *Neuroimmunology & Neuroinflammation*; consulted for Sanofi-Genzyme, Biogen Idec, MedImmune, Shire, and Alexion; received research support from Bayer, Novartis, Biogen Idec, Teva, Sanofi-Aventis/Genzyme, Alexion, Merck Serono, German Research Council, Werth Stiftung of the City of Cologne, German Ministry of Education and Research, Arthur Arnstein Stiftung Berlin, EU FP7 Framework Program, Arthur Arnstein Foundation Berlin, Guthy Jackson Charitable Foundation, and National Multiple Sclerosis of the USA. SK serves as a Deputy Editor of Journal of Neurology, Neurosurgery, and Psychiatry and an Editorial Board member of Journal of the Neurological Sciences. The remaining authors declare that the research was conducted in the absence of any commercial or financial relationships that could be construed as a potential conflict of interest.
